# P05.07. Evaluation of large scale resilience training program using complementary and alternative medicine: results from qualitative data interviews

**DOI:** 10.1186/1472-6882-12-S1-P367

**Published:** 2012-06-12

**Authors:** L Xenakis, L Hilton, R Delgado, S Libretto, J Walter

**Affiliations:** 1 Samueli Institute, Alexandria, USA

## Purpose

Military personnel are among the most at-risk populations for exposure to traumatic events and the subsequent development of psychiatric and physical illness. It has been shown that providing education and skills training in mind-body techniques may be an effective strategy for mitigation of stress-related issues. Samueli Institute has conducted a systematic program evaluation of a self-regulating skills resilience training program that rigorously appraises the program’s structure, process, and outcomes in order to determine its feasibility, acceptability, and impact on health outcomes.

## Methods

A training program designed to provide a basic education on neurological and biophysical responses to stress, and practical skills training to minimize the negative effects of stress and improve performance was given to a brigade combat team in Ft. Carson, CO at two-time points: pre- and post-deployment. Program participants were taught five core skills sets; breathing, attention, visualization, energy management, and recovery and then asked questions about their thoughts, opinions, and experiences with the training providing rich descriptions. To test the hypothesis that the resilience training program is feasible, acceptable, and effective we utilized a mixed-methods program evaluation approach.

## Results

We conducted 129 focus groups and 60 individual interviews with a total of 625 participants. Participants anticipated that breathing and recovery skills would be the most useful during deployment and with stress at home or in the work place. We will present qualitative data analysis results based on a grounded theory inquiry of a skills-based approach for stress management and resilience.

## Conclusion

The training was found to be feasible and acceptable for a military population and the majority of participants had a positive impression towards the overall training experience. Most participants expected to use what they learned and would recommend it to others. It was also suggested that the training become Army-wide or expanded to include other branches.

